# A fatal invasive *Scedosporium apiospermum* pulmonary infection in an adult patient with malignant lung adenocarcinoma

**DOI:** 10.18502/cmm.6.3.3982

**Published:** 2020-09

**Authors:** Hafize Sav, Rabiye Altinbas, Zehra Bestepe Dursun

**Affiliations:** 1 Department of Mycology, Kayseri City Hospital, Kayseri, Turkey; 2 Department of Mycology, Eskisehir City Hospital, Eskisehir, Turkey; 3 Department of Infectious Diseases and Clinical Microbiology, Kayseri City Hospital, Kayseri, Turkey

**Keywords:** Febrile neutropenia, İnvasive fungal disease, *Scedosporium apiospermum*

## Abstract

**Background and Purpose:**

*Scedosporium apiospermum* complex as a ubiquitous environmental mold is increasingly reported to cause an invasive fungal infection in immunosuppressive hosts. Herein, we present the case of an immunosuppressive 54 - year-old man who developed *S. apiospermum* complex lung infection and pulmonary adenocarcinoma.

**Case report:**

The patient had some complaints of dyspnea and cough during a neutropenic episode. The computed tomography (CT) scan of the patient revealed pleural effusion. After culturing the pleural fluid sample, the fungus was identified by microscopic examination and ITS sequencing. In addition, antifungal susceptibility testing was performed using the M38-A2 microdilution method. The minimum inhibitory concentrations of amphotericin B, voriconazole, posaconazole, and caspofungin were obtained as > 64, 0.06, 0.06, and 0.03 µg/mL, respectively. Voriconazole (administered in two doses of 6 mg/kg and a maximum of 250 mg) was preferred for treatment. The patient received antifungal treatment for 2 months; however, he was lost to follow-up.

**Conclusion:**

*Scedosporium apiospermum* complex should be considered a cause of systemic fungal infections in neutropenic patients. Furthermore, the determination of the in vitro antifungal susceptibilities of clinical strains may contribute to the development of therapeutic approaches.

## Introduction

Invasive fungal infections are on a growing trend due to the increasing number of immunosuppressed and critically ill patients.

Such risk factors as neutropenia or use of corticosteroid and cytotoxic agents play an important role in the development of invasive fungal infections [ 1 ]. The protective layers of the body, such as the mucosa and skin, are strengthened by body pH and enzymatic defenses against fungal agents. Spores and hyphae can reach the deeper tissues as a result of some invasive interventions, surgical operations, catheters, and use of the therapeutic agents of radiotherapy and chemotherapy. In this regard, the environmental fungi can cause invasive fungal infections in the host by impairing the immune system [ 2 ].

*Scedosporium* species are environmental filamentous fungi present in the soil and dirty water, which account for the development of endophthalmitis, mycetoma, osteomyelitis, and disseminated infections [ 3 - 6 ]. These species are rarely identified by the routine diagnostic mycological tests and are difficult to treat, especially when considering the optimal treatment, intrinsic resistance to systemic antifungals (e.g., fluconazole and amphotericin B) reduces the success rate of the treatment [ 7 ].

Based on the evidence, *S. prolificans* is resistant to caspofungin [ 8 ]. However, some studies have reported voriconazole as an effective option for the invasive fungal infections caused by *Scedosporium* species [ 9 , 10 ]. Limited clinical experience exists regarding the diagnosis of fungal infections caused by *Scedosporium* species. Therefore, in the present case report, we aimed to report the development of invasive *S. apiospermum* pulmonary infection in an adult patient with malignant lung adenocarcinoma. In addition, it was aimed to present data related to the diagnosis of this infection, alongside a pleural effusion, and investigate the in vitro antifungal susceptibility of the given species.

## Case report

A 54-year-old male patient with pneumonia prediagnosis referred to our hospital with such complaints as the shortness of breath and fever (day 0). The laboratory study revealed the white blood cell count of 8.540 mm^3^, C-reactive protein level of 112 mg/L, and hemoglobin level of 10.6 g/dL. Among the radiological examinations, positron emission tomography indicated parenchymal mass in the left lung lobe. In addition, metastatic lymph nodes were detected in the left hilar region. The pathological examination of the left lung as a result of bronchoscopic biopsy (day+7) led to the diagnosis of adenocarcinoma. Adenocarcinoma was found to be at stage III, accompanied by the spread of the bronchoalveolar lavage. Following the diagnosis, the patient was put on paclitaxel plus carboplatin chemotherapy and simultaneous radiotherapy.

**Figure 1:**
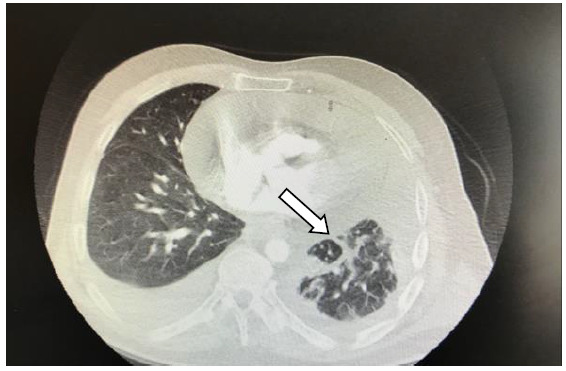
Presence of a mass lesion a year ago in the left lung lobe

**Figure 2:**
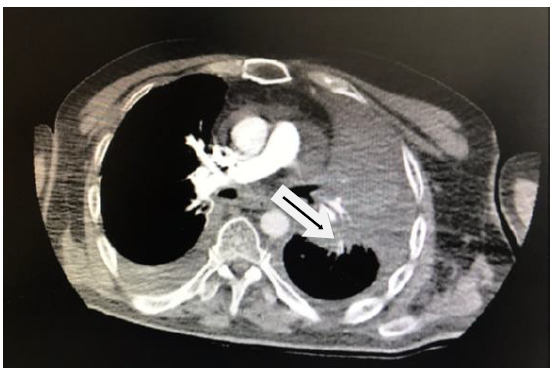
Presence of a mass lesion in the left lung lobe

The patient did not receive steroid treatment; however, he developed neutropenia during the chemotherapy (absolute neutrophil count: 279/µL, day +72) and complained of coughing. The thoracic CT revealed 2.5 cm-thick pleural effusion in the left hemithorax. In addition, a mass lesion reaching 3.5 cm in thickness was observed in the left lobe apicoposterior segment, which resulted in the loss of calibration in the vascular structures. The comparison of the mass size with that of the previous year was indicative of the regression of the lesion size. However, pleural effusion reaching up to 7 cm was observed in the thickest area extending to the left apex (figures 1 & 2).

While on the treatment, the patient's complaints of coughing and shortness of breath increased (day +92). Accordingly, to drain pleural effusion, a chest tube was placed, and chemotherapy was continued. Fungal growth was detected in the pleural fluid culture. Thoracic CT scan showed no changes in the basic mass lesion. Furthermore, changes in lesion densities were consistent with those occurring at the beginning of cavitation. The recovery of a hyaline mold by culturing a pleural mai was evaluated, and the patient was classified as a proven case according to the European Organization for Research and Treatment of Cancer/Mycoses Study Group [ 11 ].

The duration of neutropenia was calculated for the patient with an absolute neutrophil count of < 500/μL, at least once during the 3 months before the onset of invasive pulmonary disease. Voriconazole (in two doses of 6 mg/kg and a maximum of 250 mg) was preferred for treatment according to the results of antifungal susceptibility testing.

**Mycological examination**

**Figure 3:**
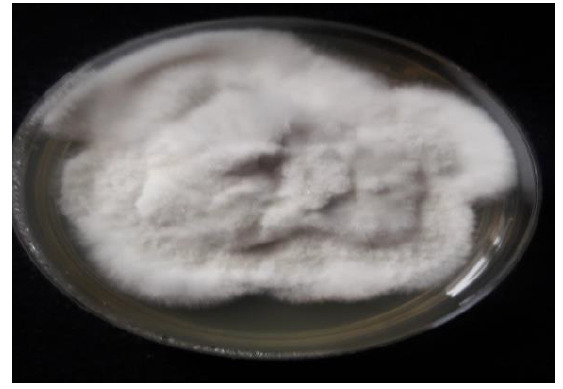
Colony of *Scedosporium apiospermum* complex on Sabouraud dextrose agar medium (The colony appearance is cottony and velvety, with the color being changed from white to grey.)

**Figure 4:**
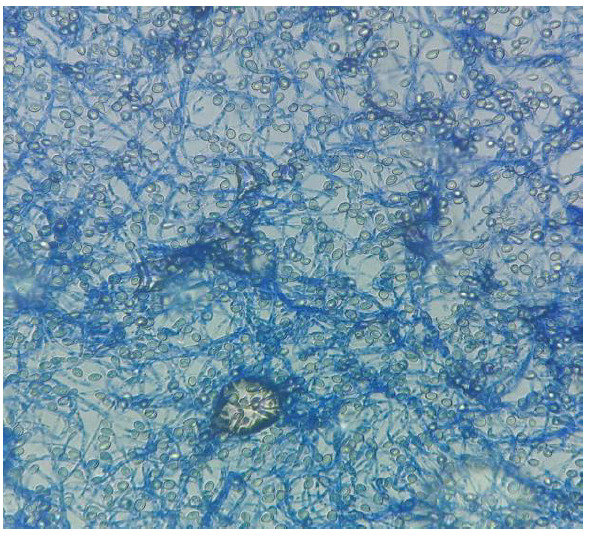
Microscopic examination of the slide culture stained with lactophenol cotton blue staining and branching septated hyphae and ovale sesile conidia

Pleural fluid was cultured on two Sabouraud dextrose agar (SDA; Oxoid, England) media with cycloheximide and without cycloheximide slants and then incubated at 25°C and 37°C for 7 days. This led to the detection of rapidly growing fungal colonies. Initially, the colony appearance was cottony and velvety with the color being changed from white to grey (Figure 3). Septate hyaline hyphae were observed in the culture by microscopic examination with lactophenol. Conidia were observed to have an oval shape. They were placed onto conidiophore as small groups or solitarily (Figure 4).

**Molecular Identification**

The DNA analysis of the clinical species was performed at a private laboratory (Bioeksen) using polymerase chain reaction (PCR) products, primers, and ABI Prism 377 DNA Sequencer (Applied Biosystems, USA). The identification of *S. apiospermum* complex was finally accomplished based on the sequencing of the PCR amplicons of the internal transcribed spacer 1 and internal transcribed spacer 2 [ 12 ]. The data of sequence analysis were analyzed using the “National Center for Biotechnology Information (Bethesda, ABD)” BLAST system ( http://www. ncbi.nlm.nih.gov/BLAST/). The GenBank accession number was determined as MT338936.

**Antifungal susceptibility**

The stock solutions of amphotericin B, voriconazole, posaconazole (Sigma, St. Louis, MO, USA), and caspofungin (Merck Sharp & Dohme BV, Haarlem, The Netherlands) were prepared using the M38-A2 reference microdilution method [ 13 ]. The serial two-fold dilutions of each antifungal were prepared in RPMI 1640 medium with L-glutamine and without sodium bicarbonate (Sigma Chemical Co., USA) and then buffered to a pH of 7.0 with 0.165 M MOPS (Sigma Chemical Co., USA).

Fungal suspensions were adjusted spectropho- tometrically to an optical density of 0.15-0.17 (68-70% transmittance) for *S. apiospermum* complex. These suspensions were diluted to 1:50 in RPMI. Each well of the microtiter plates was inoculated with 0.1 mL of the inoculum fungal suspension and then incubated at 35°C. The minimum inhibitory concentrations (MICs) were read at 48 h. Susceptibility testing revealed the MIC values of > 64, 0.06, 0.06, and 0.03 μg/mL for amphotericin B, voriconazole, posaconazole, and caspofungin, respectively. Consequently, voriconazole (administered in two doses of 6 mg/kg and a maximum of 250 mg) was preferred for treatment according to these results.

**Ethical Statements**

As this manuscript is a case report, ethical rules were not violated; therefore, there was no need to request any ethical certificate.

## Discussion

*Scedosporium* infections are rare conditions that generally cause disseminated infections in immunocompromised patients or local infections in immunocompetent patients [ 5 ]. The most notable risk factors for immunocompromised patients are prolonged neutropenia and corticosteroid therapy. Howden et al. reported the incidence of *S. prolificans*-induced disseminated infection in a patient after a bone marrow transplantation [ 6 ]. Furthermore, in another case report, *Scedosporium* was isolated from the culture of a clinical sample of a patient being treated for asthma with low- dose corticosteroids [ 9 ]. In an epidemiological study, Rodriguez-Tudela et al. investigating *P. boydii* infections, introduced neutropenia as a notable risk factor for the development of *Scedosporium* species infections [ 14 ]. Accordingly, in the present case report, the duration of neutropenia in the patient was proposed to be dependent on the usage of chemotherapeutic agents, which are known to play a role in the development of *Scedosporium* fungal infections.

Antifungal susceptibility tests were performed using the reference test method as *Scedosporium* species have intrinsic resistance to many antifungal agents. Based on the results, the isolated *S. apiospermum* complex sample had higher MIC values for amphotericin B and fluconazole, while showing lower MIC values for voriconazole, posaconazole, and caspofungin. There is no well-established standard of care for the management of these fungal infections. The reported MIC values of antifungals against the *Scedesporium* species are higher in many in vitro studies.

Based on the evidence, triazoles are more effective against *S. apiospermum* and *P. boydii* infections [ 15 , 16 ]. In our case, antifungal susceptibility was studied and following the results of this test, voriconazole (administered in two doses of 6 mg/kg and a maximum of 250 mg) was initiated. However, the patient did not respond to antifungal treatments, and he was lost to follow-up.

## Conclusion

As the present study indicated, *S. apiospermum* complex had the ability to cause an invasive pulmonary fungal infection in an adult patient with malignant lung adenocarcinoma. Based on the evidence, neutropenia is a risk factor for the development of this disease. Our patient was subjected to voriconazole as primary therapy. Although the patient was given appropriate antifungal therapy, the treatment was not successful. Consequently, in order to improve the mortality rate, it is necessary to establish rapid diagnostic methods and efficient therapeutic approaches.

## Author’s contribution

Z. B. D. analyzed and interpreted the clinical data, H. S. wrote the manuscript, and R. A. performed routine laboratory examinations.

## Conflicts of interest

The authors declared no potential conflicts of interest with respect to the research, authorship, and/or publication of this article.

## Financial disclosure

This research received no specific grant from any funding agency in the public, commercial, or not-for-profit sectors.
